# Early Human Prostate Adenocarcinomas Harbor Androgen-Independent Cancer Cells

**DOI:** 10.1371/journal.pone.0074438

**Published:** 2013-09-25

**Authors:** Rita R. Fiñones, Jo Yeargin, Melissa Lee, Aman Preet Kaur, Clari Cheng, Paulina Sun, Christopher Wu, Catherine Nguyen, Jessica Wang-Rodriguez, April N. Meyer, Stephen M. Baird, Daniel J. Donoghue, Martin Haas

**Affiliations:** 1 Department of Mechanical and Aerospace Engineering, University of California San Diego, La Jolla, California, United States of America; 2 Moores UCSD Cancer Center, University of California San Diego, La Jolla, California, United States of America; 3 Department of Pathology, University of California San Diego, La Jolla, California, United States of America; 4 Division of Biological Sciences, University of California San Diego, La Jolla, California, United States of America; 5 Department of Chemistry and Biochemistry, University of California San Diego, La Jolla, California, United States of America; Innsbruck Medical University, Austria

## Abstract

Although blockade of androgen receptor (AR) signaling represents the main treatment for advanced prostate cancer (PrCa), many patients progress to a lethal phenotype of “Castration-Resistant” prostate cancer (CR-PrCa). With the hypothesis that early PrCa may harbor a population of androgen-unresponsive cancer cells as precursors to CR-recurrent disease, we undertook the propagation of androgen-independent cells from PrCa-prostatectomy samples of early, localized (Stage-I) cases. A collection of 120 surgical specimens from prostatectomy cases was established, among which 54 were adenocarcinomas. Hormone-free cell culture conditions were developed allowing routine propagation of cells expressing prostate basal cell markers and stem/progenitor cell markers, and which proliferated as spheres/spheroids in suspension cultures. Colonies of androgen-independent epithelial cells grew out from 30/43 (70%) of the adenocarcinoma cases studied in detail. Fluorescence microscopy and flow cytometry showed that CR-PrCa cells were positive for CD44, CD133, CK5/14, c-kit, integrin α2β1, SSEA4, E-Cadherin and Aldehyde Dehydrogenase (ALDH). All 30 CR-PrCa cell cultures were also TERT-positive, but negative for TMPRSS2-ERG. Additionally, a subset of 22 of these CR-PrCa cell cultures was examined by orthotopic xenografting in intact and castrated SCID mice, generating histologically typical locally-invasive human PrCa or undifferentiated cancers, respectively, in 6–8 weeks. Cultured PrCa cells and orthotopically-induced *in vivo* cancers lacked PSA expression. We report here the propagation of Cancer Initiating Cells (CIC) directly from Stage I human PrCa tissue without selection or genetic manipulation. The propagation of stem/progenitor-like CR-PrCa cells derived from early human prostate carcinomas suggests the existence of a subpopulation of cells resistant to androgen-deprivation therapy and which may drive the subsequent emergence of disseminated CR-PrCa.

## Introduction

Blockade of androgen receptor (AR) signaling represents the main treatment for advanced prostate cancer [1]. Nonetheless, many patients progress to a fatal phenotype of “Castration-Resistant” prostate cancer (CR-PrCa). As PrCa is heterogeneous [2], [3], we hypothesized that early PrCa may contain a population of androgen-unresponsive cancer cells that serves as precursors to CR-recurrent disease. We embarked on the identification of androgen-independent cells from PrCa-prostatectomy samples of early, localized (Stage-I) cases, contained within the prostate.

The existence of epithelial prostate stem cells is widely accepted based on the extraordinary regenerative capacity of the prostate [4]–[6]. While androgen withdrawal induces apoptosis of luminal epithelial cells, basal cells remain intact, allowing rapid regeneration upon androgen replacement and suggesting that prostate stem cells reside in the basal cell layer. Prostate luminal cells have been shown to give rise to human PrCa following over-expression of specific genes [7]. Of note, stem/progenitor cells have not been propagated in an unmodified state from early stages of CR-PrCa [8], [9]. Despite the presence of Cancer Initiating Cells (CIC) in immortal PrCa cell lines derived from metastatic PrCa [10], the role of epithelial stem/progenitor cells in the generation of prostate CIC remains elusive [11].

Current models suggest that PrCa begins with the development of prostatic intraepithelial neoplasia (PIN), becoming locally invasive adenocarcinoma, followed by metastatic androgen-dependent and, finally, androgen-independent cancer [4], [12], [13]. Using cell surface markers, the isolation of prostate CIC has been reported [14]–[16]. In mice, the introduction of constitutively active AKT kinase in Sca-1-enriched prostate epithelial cells resulted in tumor initiation [17] and, in human cells, over-expression of AKT, ERG and AR in luminal cells generated prostate cancer [7]. In specimens of human Stage I prostate cancers, 0.1% of cells expressed prostate cancer stem/progenitor-like cell markers, including CD44, CD133, CK5/14 and integrin α2β1 [18], [19]. Importantly, primary PrCa cells can be immortalized by hTERT gene-transfer, and exhibit high self-renewal potential [9], [20].

We report here the propagation of CIC directly from Stage I human PrCa tissue without selection or genetic manipulation. A collection of 120 surgical prostatectomy specimens was established, among which 54 samples were adenocarcinomas. Hormone- and serum-free cell culture conditions were developed to allow the routine establishment of cells that express prostate basal cell markers and stem/progenitor cell markers, and which proliferated as spheres/spheroids in suspension cultures. Additionally, carcinoma-derived PrCa cells were successfully propagated from 30/43 of these adenocarcinoma cases. Of these, PrCa cell cultures derived from 22 adenocarcinoma samples were further examined by orthotopic xenografting and found to generate typical prostate cancers, or undifferentiated tumors, respectively, in orthotopic xenograft models in hormonally intact and castrated SCID mice. The cultured cells are “Castration-Resistant” and androgen-independent cancer cells and thus satisfy *in vitro* and *in vivo* criteria of CIC. CR-PrCa cells propagated as described here can now be used to analyze mechanisms of self-renewal [21]–[23], changes in gene expression, selection for novel mutations, metastatic progression, and therapeutic responses.

## Methods

Experimental methods are presented in Methods S1.

### Ethics Statement

All human samples were anonymously coded and obtained according to UCSD IRB#130397. All procedures performed in the work described were meticulously described to the UCSD-IRB and were presented to, reviewed and authorized by the full UCSD Institutional Review Board. A detailed Informed Consent package that was specially written for this study was extensively explained to each patient donating tissue, prior to the clinical procedures and, when understood by and agreed on by each patient, was duly signed by each patient. These records are on file in the respective clinical Departments. The entire process was fully documented in hardcopy and digitally as part of the clinical patient data; these records are saved. The research team has not had access, currently does not have access and in the future will not have access to any identifying or clinical details nor follow-up data of the patients. There was no personnel overlap between the clinical and the research teams, activities that take place in separate buildings on the UCSD Campus in La Jolla. Consented patients were entered into the study consecutively, without any categorization, whether on socioeconomic, racial, religious, nutritious, environmental exposure or similar bases. All tissue samples studied were donated at the conclusion of clinical prostatectomy procedures. These procedures are, and have been meticulously adhered to and have been authorized by the UCSD-IRB Ethics Committee. The procedure protocols are, and have been re-visited and re-authorized by the UCSD-IRB Committee on an annual basis. Patient privacy and consents are taken very seriously at UCSD.

Ethical use of immune deficient mice for the tissue-recombination transplantation and the orthotopic transplantation of ER-obtained human prostate cancer cells has been scrutinized and authorized by the UCSD IACUC authorities by means of the animal protocol # S07410. The IACUC Committees have been presented with all aspects of the experiments, including the source of the human cells transplanted, the informed consents, etc. and have authorized the work on ethical animal rights and on humanitarian bases.

## Results

### Growth of primary prostate epithelial cultures

**Figure 1:**
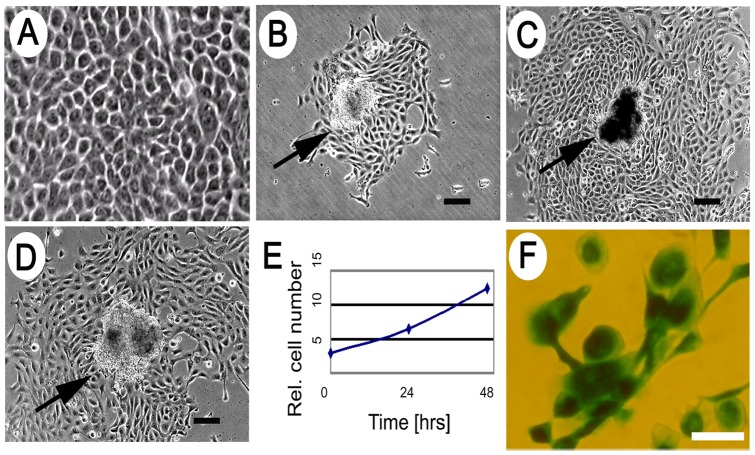
Epithelial morphology of primary Stage I PrCa cells. (**A**) Typical cellular morphology is shown, with a majority of the cells generated from primary PrCa samples displaying a tight, honeycomb morphology. (**B–D**) Examples are shown (see arrows) of semi-adherent clusters, termed Cancer Cell Clusters (CCCs), originating in individual colonies. Scalebar  = 100 μm. (**E**) Growth of PrCa cells from a single CCC at 3 time points after first observation of colony: 0, 24, and 48 h, quantified by image analysis. (**F**) Senescence assay of cells at 8^th^ passage, showing positive staining for senescence-associated β-galactosidase. Scalebar  = 50 μm.

After screening numerous media, growth factors, extracellular matrix and environmental combinations, PrCa cells were propagated from human Stage I prostate carcinomas as described (see Methods S1), using synthetic medium lacking serum or androgens. Single epithelial cell colonies were observed within 3 d after plating into growth medium (“Medium 6^+++^”), and at 7–9 d the number of colonies was stable. These cultures propagated to form epithelial, honeycomb-like cells, which maintained close cell-cell contact (Fig 1A), and which exhibited a small, compact group of cells provisionally designated as a Cancer Cell Cluster (CCC) (Fig 1B–D). PrCa cell colonies initially divided with a doubling time of ∼20 h (Fig 1E). Cultures were passaged until about the 8^th^ passage or ∼30 population doublings, at which point cells began to adopt a large, flattened morphology and were positive for senescence-associated β-galactosidase (Fig 1F). The results of 43 out of 54 cases diagnosed as prostate adenocarcinomas of Gleason Score ranging from 6–9 are summarized (Table S1). Table S1 also lists the number of epithelial colonies obtained from each of the 30 adenocarcinoma samples that yielded epithelial colonies. The remaining 13 adenocarcinoma samples yielded no colonies and were not further studied.

As controls, samples obtained by transurethral resection and diagnosed as benign prostatic hyperplasia (BPH) were processed and grown identically to the adenocarcinoma samples. Only 2 of 50 samples diagnosed as BPH cases generated low numbers (3–10) of poorly-growing epithelial colonies.

The data presented here demonstrate the existence of cells that can be directly propagated from Stage-I prostate cancer in defined media lacking serum or androgens. The propagation of androgen-independent and cancer-inducing cells from early (Stage I) human prostate adenocarcinoma has not been previously described.

### Cell-marker expression by PrCa cells

**Figure 2:**
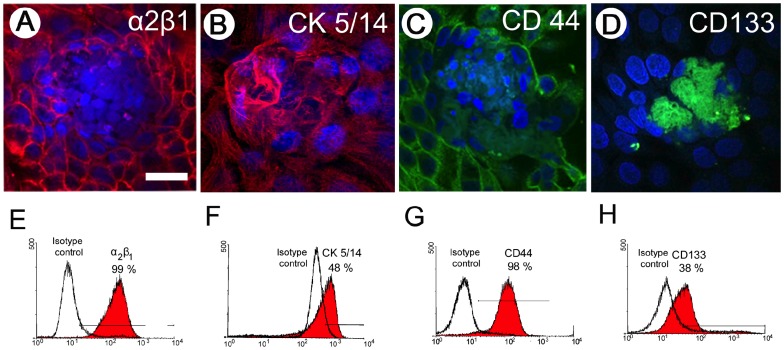
Prostate stem/progenitor cell marker expression by confocal microscopy and flow cytometry. Prostate CCC and surrounding prostate epithelial PrCa cells expressed: (**A**) integrin α2β1 (red); (**B**) high-molecular weight cytokeratin (CK5/14) (red); (**C**) CD44 (green); and (**D**) CD133 (green). Nuclei detected with DAPI (blue). Flow-cytometry of same markers is presented for pooled PrCa cells and CCCs: (**E**) integrin α2β1 (∼99% positive); (**F**) CK5/14 (∼48% positive); (**G**) CD44 (∼98% positive); and (**H**) CD133 (∼38% positive). Expression of CD133 decreased rapidly in later colony transfers, which correlates with progressive dilution of CD133^+^ CCCs, relative to more rapidly proliferating CD133^lo/−^ PrCa cells.

Early-passage PrCa cells expanded from single colonies were assayed by indirect immunofluorescence for prostate progenitor cell markers integrin α2β1, CK5/14, CD44 and CD133. Early passage cultures contained both visible cell clusters and an expanding halo of epithelial cell progeny expressing integrin α2β1, CK5/14 and CD44 (Fig 2A-C). CD133 expression was limited to cells in the tight clusters (Fig 2D) but was soon lost after passaging.

By flow cytometry, PrCa cells expressed integrin α2β1 (Fig 2E), high molecular weight (basal cell) cytokeratin CK5/14 (Fig 2F), and CD44 (Fig 2G). CD133 was expressed in early passage cultures (Fig 2H), but expression was lost upon outgrowth into sheets of epithelial cells. Hence, the range of CD133 expression by our PrCa cell cultures decreased rapidly from about 50% in freshly explanted colonies to undetectable levels at culture passage 2 and above.

PrCa cell colonies were also assayed for prostate stem/progenitor and differentiation markers. Double-staining for p63, CK8/18, and c-kit, together with CD44, was performed. The basal cell marker p63 was mostly localized to the nucleus in PrCa/CCC cells (Fig. S1A), as is commonly found in normal prostate basal cells. Though p63 tends to be underexpressed in adenocarcinomas [24], some cultured prostate carcinoma cell lines have been shown to express nuclear p63 [25]. Additionally, cytoplasmic p63 has been associated with prostate cancer mortality [26]. Cultured PrCa/CCC cells also expressed the prostate differentiation (luminal cell) marker CK8/18 (Fig. S1B), and c-kit (Fig. S1C), which co-localized with CD44. Cultured PrCa cells expressed neither chromogranin A nor PSA. The surprising lack of PSA expression was confirmed using 8 different PSA-specific antibodies on 22 different PrCa cell cultures (data not shown).

### Expression of the Aldehyde Dehydrogenase (ALDH) stem cell-specific enzyme

**Figure 3:**
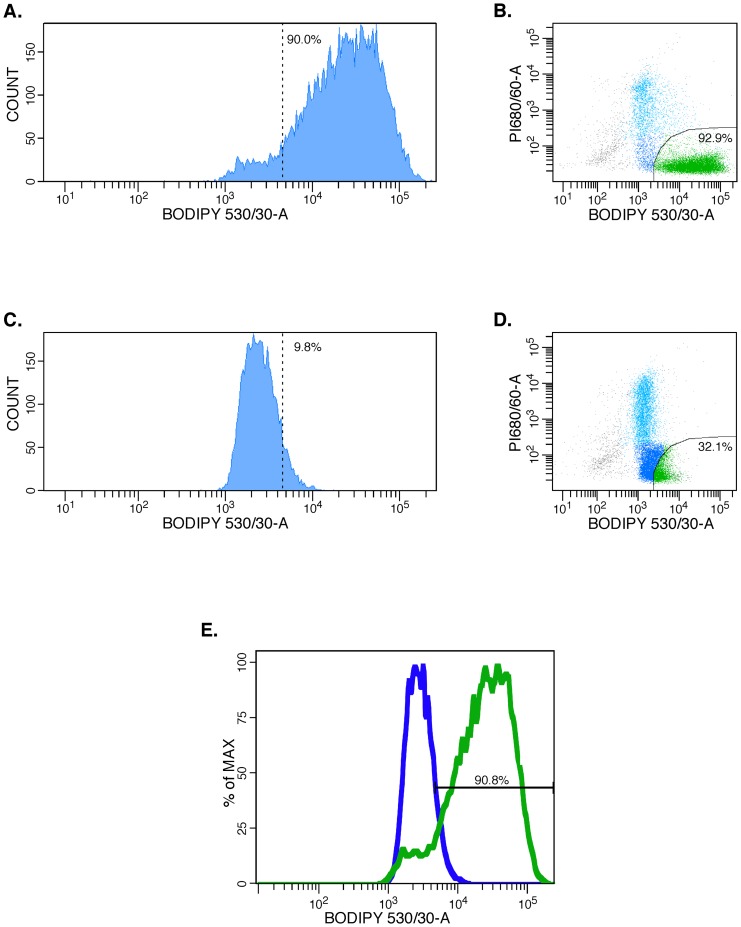
Representative Flow Cytometry (FACS) assay of ALDEFLUOR testing for ALDH-expression by PrCa cells. PrCa #109 cells, tr.1 were trypsinized, reacted with the ALDH1A1 substrate BODIPY-aminoacetaldehyde (BAAA). The ALDH inhibitor DEAB was added to a control tube of the same cells to show the specificity of ALDH1-activated BODIPY-fluorescent detection. The median fluorescent ALDH1-signal of 3×10^5^ units was inhibited ∼200× by the DEAB inhibitor. 90.0% ALDH1-bright cells (**A and C**) were reduced to 9.8% in the presence of the ALDH DEAB inhibitor (**B and D**). The relevant dot-plots are shown on the right, and a contour plot comparing the ALDH-activated fluorescence and that of cells in which the reaction was inhibited by DEAB is shown below (**E**).

ALDH, a detoxifying enzyme responsible for oxidizing aldehydes to carboxylic acids, has served as a functional marker [27], [28] for the presence of stem cells and CIC in a wide variety of cancers [29]–[31]. In breast cancer CIC, the ALDH1A3 isotype predominates and is predictive of metastasis [32], while in prostate cancer the ALDH7A1 isotype predominates [33]. In our cultured PrCa cells, ALDH was strongly expressed (90.0% for Pr #109), but was reduced to 9.8% in the presence of the ALDH-specific inhibitor diethylaminobenzaldehyde (DEAB) (Fig 3). MCF7 cells were used as an ALDH-negative control in these experiments (data not shown). With increasing passage number, ALDH expression in PrCa cells decreased, reaching less than 1% after 6 passages (data not shown).

Additionally, expression of the ALDH7A1 isotype was demonstrated by indirect immunofluorescence in 12 different cultured PrCa cell cultures tested (Fig. S2). Expression of the isotype ALDH7A1 in PrCa cells cultured here is consistent with its reported expression in human PrCa and in immunohistology sections of human PrCa and their metastases [33].

### Cultured PrCa cells readily generate anchorage-independent spheres/spheroids in Matrigel cultures

**Figure 4:**
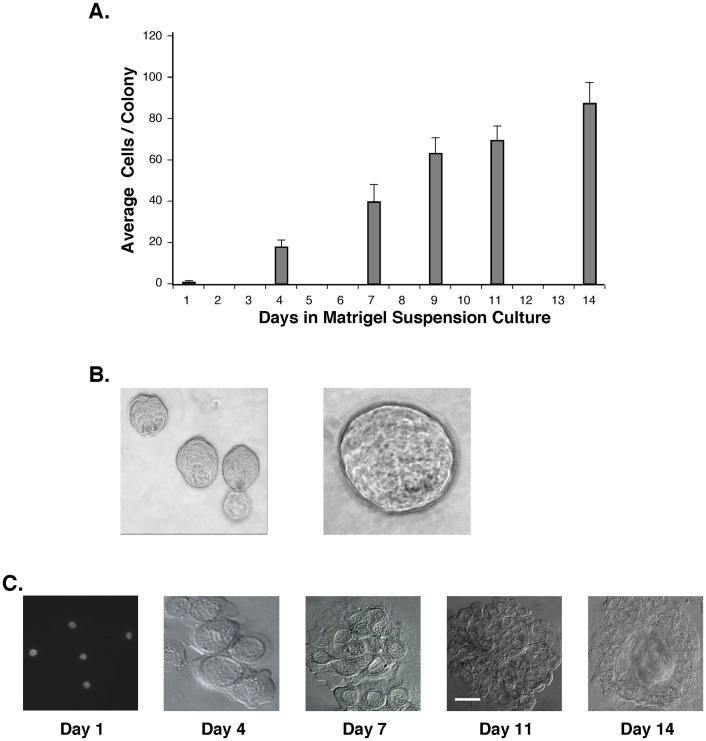
High-frequency self-renewal of PrCa cell spheroids in Matrigel suspension cultures. Low passage, adherent PrCa cells (#76, tr 2) were trypsinized and suspended in Matrigel in medium 6^+++^. Suspension cultures were grown for 14 days and spheres of replicate cultures were harvested on days 1, 4, 7, 9, 11 and 14. Cultures were digested with dispase, pelleted on microscope slides by cytocentrifugation and fixed with methanol. (**A**) The size of the spheres was determined by counting the cells of >200 stained spheres at each time-point. (**B**) Early and late time-point spheres were photographed under an inverted microscope. (**C**) Representative harvested spheres growing 1, 4, 7, 11 and 14 days in Matrigel cultures, and deposited on microscope slides for imaging, counting and antibody staining.

In contrast to differentiated cells, normal and cancer-initiating stem/progenitor cells proliferate to form spheres/spheroids under anchorage-independent culture conditions [34]. Adherent PrCa cells were trypsinized to single cells, passed through a 40 µm strainer, and 10^4^ cells plated in Matrigel suspension cultures. This resulted in the generation of growing spheres in Matrigel by >90% of the suspended single cells (Fig 4), which was independent of the Gleason Score (6–9) of the cancer donors. After staining cell nuclei with DAPI (4′,6-diamidino-2-phenylindole), the number of cells per growing sphere was quantitated as 88±9.7 cells per colony (Fig 4A). Examples of spheres in Matrigel suspension culture are shown (Fig 4B) as are spheres deposited on glass microscope slides by cytocentrifuge (Fig 4C). Spheres persisted in the expression of ALDH1, CD44, Integrin α2β1, SSEA4 and TERT (not shown). With successive transfers of the adherent PrCa cells at a 1∶3 subculture ratio, a decreasing fraction generated Matrigel spheres, with <0.1% retaining this capacity by passage 7.

### CR-PrCa cells express telomerase reverse transcriptase (TERT) while normal human prostate epithelial cells are TERT-negative

**Figure 5:**
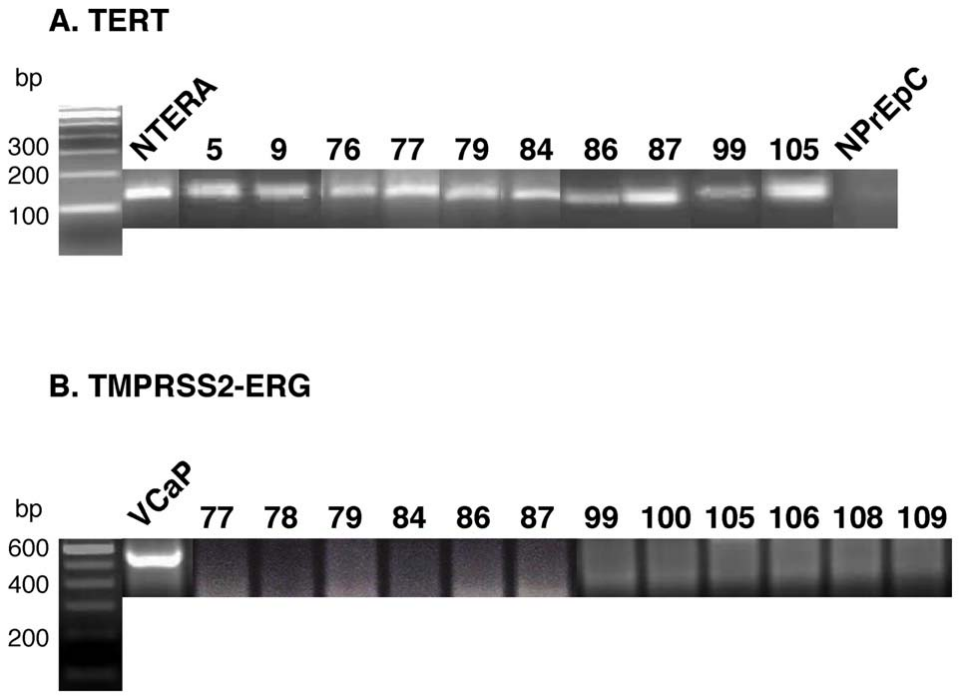
RT-PCR for Expression of TERT and TMPRSSG-ERG Fusion mRNAs. (**A**) **TERT**. RT-PCR amplification of mRNA isolated from PrCa cells for the expression of the Telomerase Reverse Transcriptase (TERT) enzyme. All of the 30 PrCaCell cultures tested expressed TERT while NPrEp cells grown from three young donors did not express the TERT gene. The immortal NTERA (normal human teratoma) cell line was used as TERT-positive control. (**B**) **TMPRSSG-ERG**. RT-PCR results of PrCaCell cultures fail to show evidence of a TMPRSS2-ERG fusion mRNA, while cells of the human vertebral metastasis prostate cancer cell line VCaP do express the fusion mRNA.

TERT expression by PrCa cells described here was assayed by Reverse Transcriptase-Polymerase Chain Reaction (RT-PCR) amplification (Fig 5A). All 30 adenocarcinomas that yielded CR-PrCa cultured cells shown in Table S1 expressed TERT RNA. RNA extracted from the human teratoma cell line NTERA served as a TERT-positive control, whereas normal human prostate epithelial cells (NPrEp) derived from young donors were TERT-negative (n = 3).

### Cultured CR-PrCa cells do not express detectable levels of the TMPRSS2-ERG fusion RNA

It remains unclear whether expression of the TMPRSS2-ERG fusion mRNA in a prostate cancer cell serves as a major driver for its tumorigenic features [33], or whether the fusion event occurs *as a result of* an androgen-responsive tumor cell state, as suggested by induction of the fusion event in normal prostate epithelial cells in response to androgen [35], [36]. Regardless, of the 30 independent PrCa samples cultured, all were negative for expression of TMPRSS2-ERG fusion mRNA by RT-PCR (Fig 5B). Since the TMPRSS2-ERG fusion RNA may be difficult to detect [37], we used multiple amplification oligo sets and the highly sensitive MyTaq amplification kit (BioLine USA, Taunton, MA) (Table S2). The human cell line VCaP, which expresses a TMPRSS2-ERG fusion mRNA, was confirmed as expressing the fusion transcript using only 1 ng of isolated cellular RNA (Table S2). The amplified fusion mRNA of VCaP cells was confirmed by sequencing of the amplification DNA product [38] (not shown). In summary, CR-PrCa cells cultured here do not express detectable TMPRSS2-ERG fusion RNA.

### Orthotopic engraftment of PrCa cells

**Figure 6:**
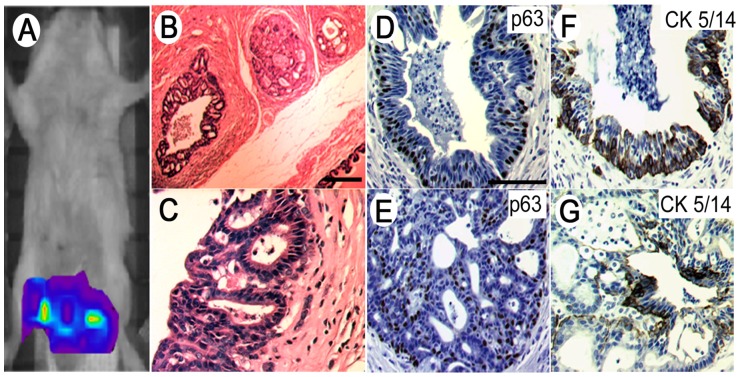
Orthotopic xenografting of PrCa/CCC cell cultures into the anterior prostate of recipient SCID mice recapitulated histological features of prostate adenocarcinoma. (**A**) *In vivo* imaging of EGFP-labeled PrCa/CCC cells engrafted in the anterior prostates of recipient SCID mice shows localization of the grafts two weeks after grafting into the anterior prostate capsules. (**B**) Hematoxylin-eosin staining of tumor growth in prostate and urogenital organs. (**C**) Higher magnification of cribriform glands suggests initial development of invasive prostate cancer. Both simpler and more complex glands were observed. (**D**) Proliferation marker p63-stained basal cell layer of complex glands. (**E**) p63 expression appears disorderly in histologically higher grade glands. (**F**) Simpler glands express the marker CK5/14, whereas (**G**) glands that appear to be progressing to cribriform prostate cancer only sporadically express high molecular weight cytokeratin.

Decreasing numbers of PrCa cells were engrafted directly into the anterior prostate of recipient SCID/Beige mice and into mice surgically and chemically castrated. In some experiments, the PrCa cells were first labeled with EGFP by infection of the cultured cells with an MSCV retrovirus encoding EGFP [39]. *In vivo* imaging of SCID mice xenografted with EGFP-labeled cells generated a clear fluorescent signal two weeks after engraftment in the anterior prostate graft site (Fig 6A). These PrCa cells developed into a locally invasive, cribriform (gland-in-gland) pattern consistent with low Gleason Grade PrCa (Fig 6B and 6C). Increased cellularity of stroma adjacent to complex glands was observed, together with central necrosis in the lumens and a disorderly mixture of basal and cuboidal epithelial cells (Fig 6D–G). Interestingly, simple tumor glands expressed p63 in their basal cell layer (Fig 6D), while in histologically more complex glands, p63 expression by basal cells was reduced and disorderly (Fig 6E).

The histologic appearance of a local cancer induced in a SCID mouse orthotopically xenotransplanted with 200 PrCa cells is compared with that of the donor Stage I cancer tissue sample (Fig. S3). Both tumors were composed of small glands in which cells with prominent nucleoli are frequent. Gland structures were often back-to-back or gland-within-gland formations. PrCa cell-induced cancers in intact SCID recipient mice presented a histological appearance that was indistinguishable from the cancer of origin. In castrated mice, poorly differentiated and widely disseminated tumors resulted. In contrast, up to 10^5^ normal human prostate epithelial cells (n = 3) that were propagated identically and transplanted orthotopically into the anterior prostates of SCID and castrated SCID mice produced no tumor growth in up to 30 weeks.

## Discussion

Currently, Stage I prostate adenocarcinoma is considered to be hormone-dependent and hormone-responsive; typically, these cancers are treated by androgen-deprivation therapies (ADT). Many patients progress to the lethal phenotype of castrate-resistant (CR) PrCa, possibly implying the “switching” of cancer cells from hormone-dependence to hormone-independence. Alternatively, minor populations of cells may have qualities that allow them to survive ADT with the potential of propagating recurrent CR-primary or metastatic disease.

In this study, we have propagated “Castration Resistant” epithelial cells directly from Stage-I human prostate cancer tissue with Gleason Scores ranging from 6 to 9 (Table S1). Using the conditions described, colonies of androgen-independent epithelial cells grew out from 30/43 cases (70%) of adenocarcinomas, 22 of which were studied by orthotopic xenotransplanation in SCID mice. PrCa cell colony numbers typically ranged from ∼40 to 500 per 10^7^ input cells (50 mg of starting cancer tissue), a frequency of 0.0004 to 0.005%, although some of the colony-producing carcinomas generated very few colonies (Table S1).

Colonies of adherent PrCa cells proliferated in the absence of androgen and serum, but did require bFGF, EGF and pituitary extract. Epithelial PrCa colonies emanated from tight centers of 10–30 cells, designated Cancer Cell Clusters (CCC) (Fig 1), which lost CD133 but expressed c-kit, p63, and E-cadherin, markers of basal cells in the prostate [40]. The markers expressed by PrCa/CCC cells – CD44, CD133, CK5/14, c-kit, integrin α2β1, ALDH, SSEA4 and E-Cadherin – are characteristic of basal cells and of prostate stem/progenitor cells [14], [41]–[43], suggesting that the CCC resembles an epithelial prostate stem cell niche.

The expression by CR-PrCa cultured cells of p63 and its partial loss on the generation of more complex *in vivo* glands following xenotransplantation (Fig 6E) suggests that the cultured CR-PrCa cells may differ only moderately from their normal counterparts in the human prostate. Nevertheless, the significant *in vivo* tumorigenicity of the cells and their unambiguous expression of TERT suggests that these CR-PrCa cells cultured from Stage-I prostate cancer represent an early manifestation of the carcinogenic process. Indeed, it will be of great interest in future experiments to compare the expression of cancer-relevant genes between Cr-PrCa cells described here and human NPrEp cells.

Orthotopic xenografting of PrCa cells (Fig 6) and resultant tumor induction confirmed the fundamental cancer phenotype of the cultured PrCa cells. Nonetheless, *in vivo* transplantation of small numbers of culture-isolated PrCa cells into SCID mice under different conditions resulted in various outcomes. First, PrCa cells transplanted in collagen or Matrigel into the anterior prostate of SCID mice produced complex, back-to-back cribriform pattern-glands that invaded the surrounding stroma, characteristic of low Gleason Grade prostate cancer. During the 12 week observation period for the othotopic xenograft experiments, metastasis was not observed. Xenotransplantation of all 22 cultured CR-PrCa cell cultures resulted in simple or more complex cancer glands (Fig 6E/F). However, the possible correlation between the Gleason Score of the original cancer (Table S1) and the complexity of xenotransplanted tumors in SCID recipients will be the subject of future inquiry. Subcutaneous transplantation of as many as 5×10^5^ PrCa cells in intact or castrated SCID mice did not result in the generation of tumors (data not shown), highlighting the microenvironment-dependent nature of the PrCa cells grown from early prostate carcinomas.

Second, transplantation using the urogenital mesenchyme (UGM) recombination xenograft model [44], [45] under the renal capsule of SCID mice (Figure S4) resulted in simple glands composed mostly of cuboidal cells that presented as basal cells expressing p63 and E-cadherin. Some of the glands developed a second layer of luminal cells that lacked p63 expression but retained E-cadherin, similar to normal prostate development [46], [47]. These results suggested that embryonal UGM in the tissue-recombination xenograft imposed normal proliferative signals on the Stage-I-derived PrCa cells [48], [49].

The lack of PSA expression by the cultured PrCa cells described here and by their orthotopically xenotransplantation-induced cancers was unexpected. Another surprise was that these cultured PrCa cells eventually senesced despite expression of TERT. PrCa cells invariably differentiated and senesced after ∼8 transfers in culture, suggesting that in the absence of an appropriate microenvironment/niche these cells were mortal. However, these same cells were apparently immortal *in vivo* in the presence of their prostate microenvironment, as evidenced by the orthotopic xenotransplantion of ∼200 PrCa cells, resulting in a cancer burden of >20 gm, equivalent to ∼25 cell doublings. Hence, self-renewal of CR-PrCa cell CICs appears to be conditional, requiring a suitable microenvironment. Indeed, the common definition of CIC embraces an immortal phenotype [50], as CICs have commonly been isolated from fully progressed and/or metastatic cancers [51]–[53]. Additionally, in castrated mice some cultured CR-PrCa cells that were orthotopically xenotransplanted into the anterior prostate of SCID mice have grown continuously until the mice had to be sacrificed as they carried an abdominal tumor burden equal to the weight of the mouse (data not shown).

The presence of CR-prostate cancer cells possessing a stem cell phenotype in early prostate cancer is consistent with recent findings [54] that document a systematic increase in PrCa stem/progenitor cells during ADT of PrCa patients, explaining how current ADT might result in an undesired expansion of a PrCa stem/progenitor cell population with therapy failure. Our findings suggest that CR-PrCa cell CICs that are present in more than half of the Stage-I patient cohort examined may relate to the therapy failure described by others [54].

Other investigators have isolated putative PrCa stem cells using a modified Hoechst 33342 dye efflux assay to isolate side-populations of enriched putative stem cells from malignant prostate tissue [55]–[58]. A second approach has examined the existence of an androgen-independent stem cell that can give rise to androgen-dependent, fully differentiated luminal cells via a transit-amplifying population positive for expression of CK5/14, CK18, CD44, ABCG2, CD133, and integrin α2β1 [14], [59], [60]. A third approach has investigated the immortalization of prostate stem cells through the forced expression of human telomerase (hTERT) [9], [20], [41], [61]. Lastly, human prostate tumor-initiating cells have been described that display stem-like properties with increased NF-kappaB activity [62]. The relationship between the PrCa cells reported here, and the cell populations described by other investigators, will require further experimental inquiry.

In summary, data presented demonstrate the presence of cancer cells, in the earliest stages of human prostate adenocarcinomas, that can be propagated in defined media lacking androgens. The unique culture conditions presented here and the relative ease of propagation of androgen-independent proliferative cell populations can be expected to stimulate further research. Significantly, these methods can be applied to small biopsy samples, allowing characterization of putative CR-prostate cancer cells and facilitating evidence-based clinical management early in the disease. Future experiments will examine gene modifications in these cells, possible drug treatments that may affect them, and further genetic alterations that may render them urogenital microenvironment-independent and potentially metastatic.

## Supporting Information

Figure S1 Prostate Stem/Progenitor Markers Expressed by PrCa Cell Colonies.
(PDF)

Figure S2 Predominant Expression of Aldehyde Dehydrogenase 7A1 by PrCa Cell Cultures.
(PDF)

Figure S3 Histologic Appearance of Local Cancer Induced by Xenotransplantation.
(PDF)

Figure S4 UGM-Mediated Tissue Recombination of PrCa Cells.
(PDF)

Methods S1 (PDF)

Results S1 (PDF)

Table S1 Culture of Representative Prostate Cancer Tissue Samples.
(PDF)

Table S2 Reverse Transcriptase PCR Condition.
(PDF)

References S1 (PDF)
