# Supplementary material for: Early Human Prostate Adenocarcinomas Harbor Androgen-Independent Cancer Cells
Source: PLoS One. 2013 Sep 25;8(9):e74438. doi: 10.1371/journal.pone.0074438 (PMC3783414; doi:10.1371/journal.pone.0074438)

Supplementary FIGURE S1.

Prostate Stem/Progenitor Markers Expressed by PrCa Cell Colonies.

Legend to Figure S1. Prostate CCC and surrounding prostate epithelial PrCa cells expressed: **(A)** p63 (red); **(B)** cytokeratin 8/18 (CK8/18) (red); **(C)** c-kit (red). For each, CD44 staining (green) is shown in the middle panel, with nuclei detected by DAPI (blue). Merged images are shown in the bottom panels. Flow-cytometry of other markers is presented for pooled PrCa cells and CCCs in Fig 2 in the main text, namely: CK5/14 (~48% positive); CD44 (~98% positive); and CD133 (~38% positive). Expression of CD133 decreased rapidly in later colony transfers, which correlates with differentiation of the cells and the progressive dilution of $CD133^{+}$ CCCs, relative to more rapidly proliferating $CD133^{lo/-}$ PrCa cells.

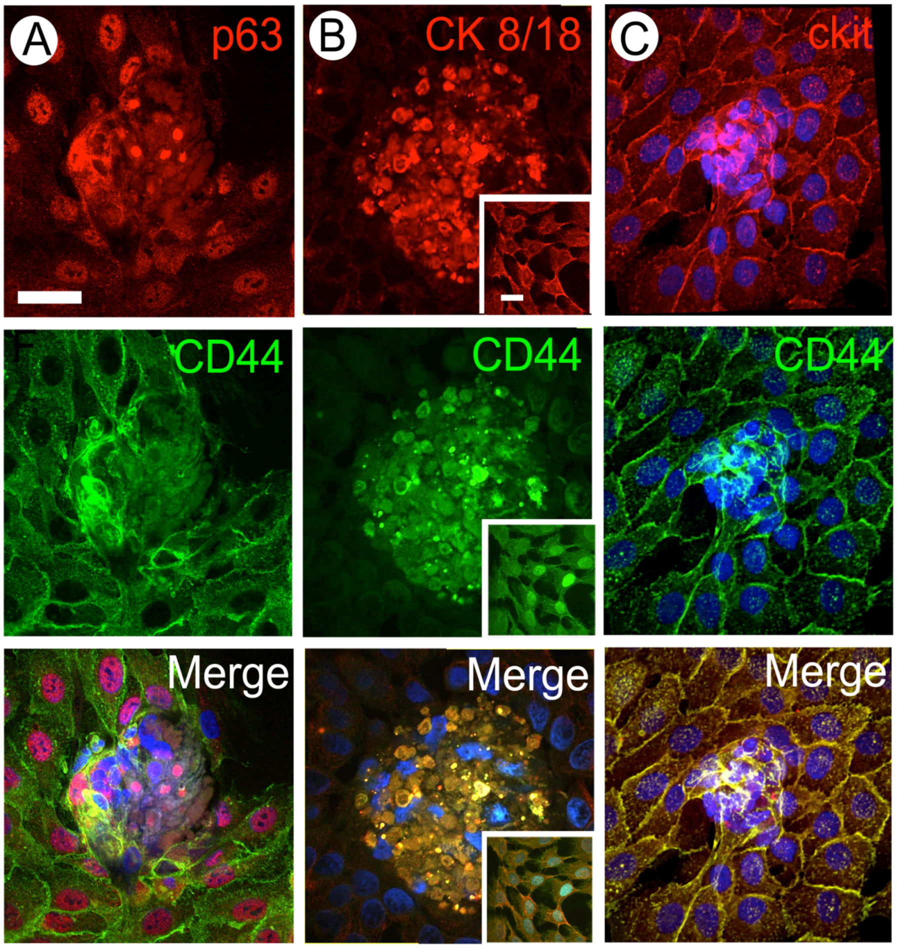

Supplement: Figure S1 — Prostate Stem/Progenitor Markers Expressed by PrCa Cell Colonies. (PDF) [file pone.0074438.s001.pdf]
