# Supplementary material for: Early Human Prostate Adenocarcinomas Harbor Androgen-Independent Cancer Cells
Source: PLoS One. 2013 Sep 25;8(9):e74438. doi: 10.1371/journal.pone.0074438 (PMC3783414; doi:10.1371/journal.pone.0074438)

Supplementary FIGURE S2.

Predominant Expression of Aldehyde Dehydrogenase 7A1 by PrCa Cell Cultures.

Legend to Figure S2. Indirect immunofluorescent staining of Prostate Cancer Cells (Pr#87) with antisera specific for ALDH isotypes 1A3 and 7A1 and non-immune rabbit control serum. PrCa cells (Pr#87) were grown on coverslips, fixed in methanol and stained with rabbit anti-bodies to ALDH1A3 (**A**), ALDH7A1 (**B**), and non-immune rabbit serum (**C**). Scale bars = 50 µm.

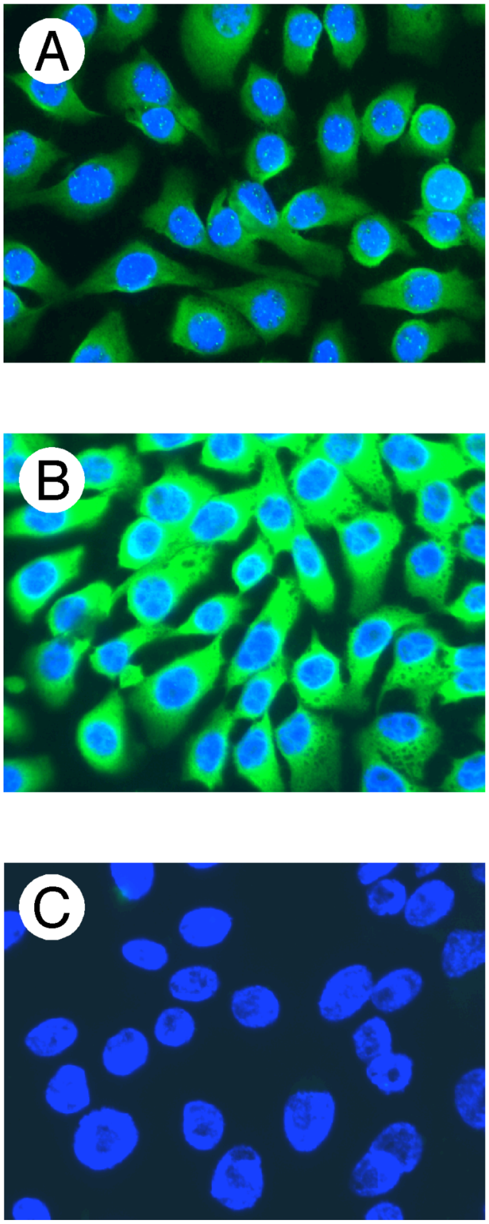

Supplement: Figure S2 — Predominant Expression of Aldehyde Dehydrogenase 7A1 by PrCa Cell Cultures. (PDF) [file pone.0074438.s002.pdf]
