# Supplementary material for: Early Human Prostate Adenocarcinomas Harbor Androgen-Independent Cancer Cells
Source: PLoS One. 2013 Sep 25;8(9):e74438. doi: 10.1371/journal.pone.0074438 (PMC3783414; doi:10.1371/journal.pone.0074438)

Supplementary FIGURE S3.

Histologic Appearance of Local Cancer Induced by Xenotransplantation.

Legend to Figure S3. (A) Micrograph of the histological appearance of local cancer generated by orthotopic xenografting of only PrCa cells (no added rat UGM cells). **(B)** Comparison with the histologic picture of the Stage I cancer of the patient donating the specific cultured prostate cancer tissue. Note the tendency to form back-to-back glands and gland-within-gland structures. Cells with nucleoli are common.

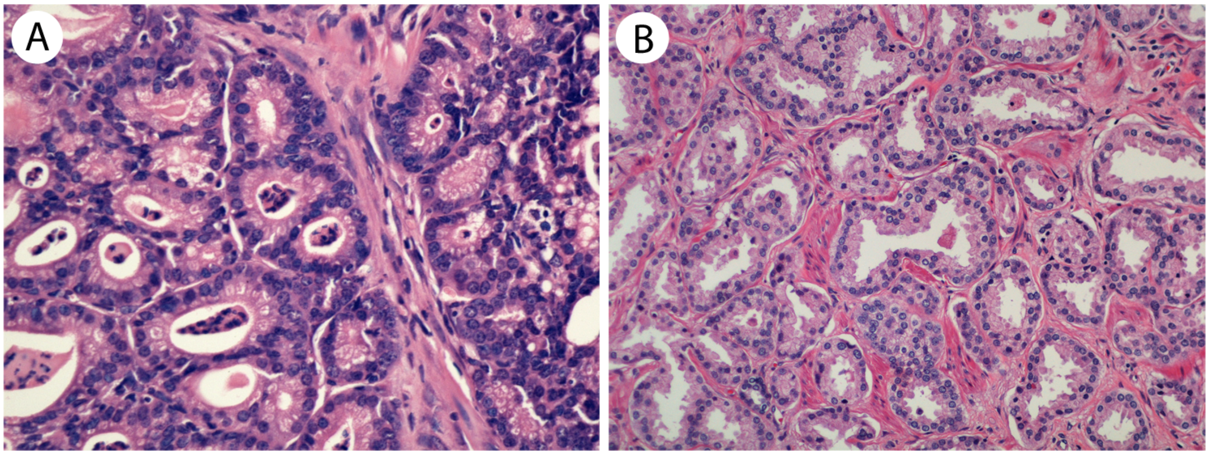

Supplement: Figure S3 — Histologic Appearance of Local Cancer Induced by Xenotransplantation. (PDF) [file pone.0074438.s003.pdf]
