# Supplementary material for: Early Human Prostate Adenocarcinomas Harbor Androgen-Independent Cancer Cells
Source: PLoS One. 2013 Sep 25;8(9):e74438. doi: 10.1371/journal.pone.0074438 (PMC3783414; doi:10.1371/journal.pone.0074438)

**Supplementary FIGURE S4.**

**UGM-Mediated Tissue Recombination of PrCa Cells.**

**Legend to Figure S4.** UGM-mediated tissue recombination of PrCa cells transplanted under the kidney capsule of SCID recipients stimulates slow-growing glands with simple epithelium. Glandular structures were observed after subcapsular renal engraftment of PrCa cells with rat embryonic urogenital mesenchyme, as seen after H&E staining (A). Cells in these glands expressed the epithelial marker E-cadherin (B) and basal cell marker p63 (C). (D) A human-specific mitochondrial antibody. Insets in B, C, and D show relative staining of positive-controls. Glands were negative for CD44, CD133, PSA, and AMACR (data not shown). Scalebars = 100 μm.

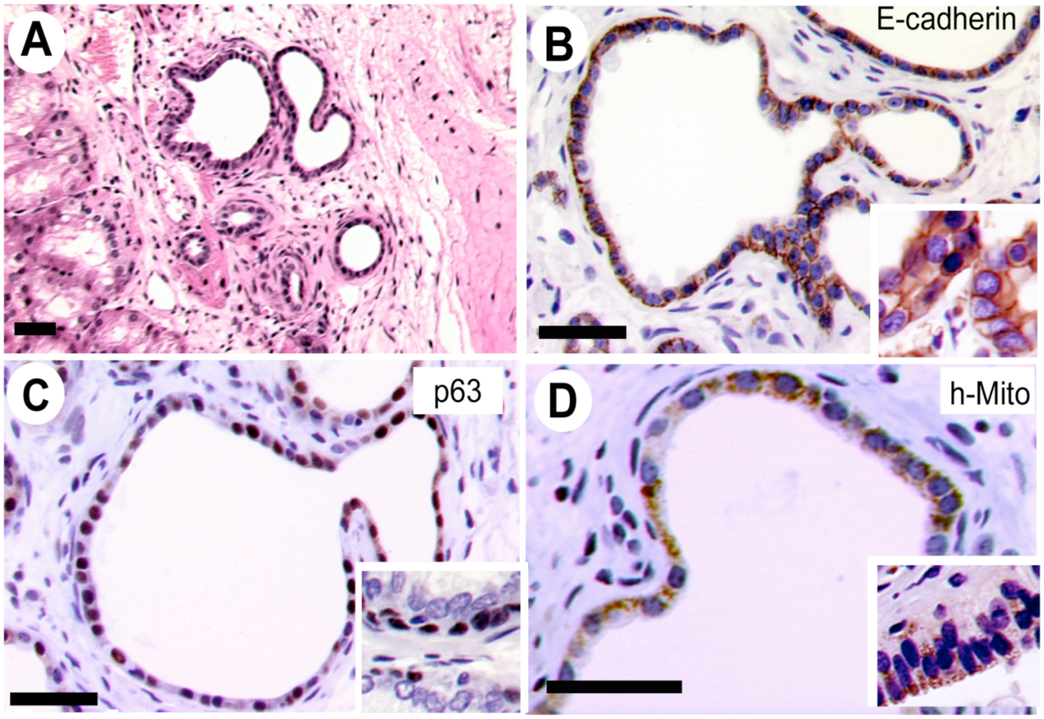

Supplement: Figure S4 — UGM-Mediated Tissue Recombination of PrCa Cells. (PDF) [file pone.0074438.s004.pdf]
