# Supplementary material for: Early Human Prostate Adenocarcinomas Harbor Androgen-Independent Cancer Cells
Source: PLoS One. 2013 Sep 25;8(9):e74438. doi: 10.1371/journal.pone.0074438 (PMC3783414; doi:10.1371/journal.pone.0074438)
Supplement: Methods S1 — (PDF) [file pone.0074438.s005.pdf]

SUPPLEMENTARY METHODS S1:

Prostatectomy Samples. Prostate cancer (PrCa) specimens were collected from PrCa tissue of patients undergoing prostatectomy. The cohort contained 54 clinical Stage I and II prostate cancers and 66 BPH cases. Sample collection was done sequentially without any selection, and was authorized by the UCSD HRPP-IRB. Frozen-section-ascertained cancers were harvested, as well as areas adjacent to presumed cancer tissue. Diagnosis was verified both by frozen sections and by routine processing of the rest of the prostate tissue for clinical histology. Among 120 prostatectomy samples (including the 54 carcinomas), cancers of Gleason Scores 6 though 9 were routinely observed. Further information describing a representative and randomly selected carcinoma subset is presented below (see Supplementary Table S1). Using the techniques described below, carcinoma-derived PrCa cells were successfully propagated from 30/43 of these adenocarcinoma cases that were studied in more detail. Of these, PrCa cell cultures derived from 22 adenocarcinoma samples were further examined by orthotopic xenografting as described below and in the main manuscript.

Cell Culture. To preclude any bias by the research team, samples were processed without knowledge of the clinical diagnosis. This resulted in a sharp division of samples yielding epithelial colonies/culture and samples that yielded no epithelial colonies (BPH cases). White nodule areas of macroscopically-presumed carcinoma tissue of primary human PrCa samples from radical prostatectomies were aseptically cut into 1-2 mm pieces within 60 min of surgery, and digested for 8 h while stirring in 150 U/ml collagenase I (Sigma-Aldrich) in growth medium at 37°C. Vials of frozen cells containing 50 mg of starting tissue in 90% FBS plus 10% DMSO (Sigma-Aldrich) were stored as live cells in liquid nitrogen. Some carcinoma nodules yielded 2-3

frozen tissue vials, with occasional samples yielding up to 30 vials of live cells. All prostatectomy tissue samples – carcinoma, BPH, or mixed – were treated identically. Tissue culture 6-well plates (Corning) were coated with 10 μg/ml laminin (Sigma-Aldrich) in PBS for 1 h at 37°C and washed twice with PBS. Prostate tumor samples were cultured in growth medium (keratinocyte serum-free medium (Gibco) with 40 mM L-glutamine (Gibco), 12.5 μg/ml gentamycin and 2.5 μg/ml amphotericin B supplemented with 10 ng/ml basic Fibroblast Growth Factor (bFGF) (R&D), 40 ng/ml EGF (R&D), 58 μg/ml Bovine Pituitary Extract (Gibco), 1 mM $CaCl_2$, and 0.025 % BSA (Sigma-Aldrich), designated "Medium $MH6^{+++}$". Cultures were incubated at 37°C in 10% CO_2, 5% O_2 and medium was changed every second day. Fast-growing, epithelial colonies grew out from tumor samples after ~ 1 week and were sub-cultured 1:3 in medium $MH6^{+++}$ onto laminin-coated cell culture surfaces upon reaching 80-90% confluence. ~90%-confluent cultures could be routinely subcultured up to 8 times resulting in $\sim 10^9$ cells from a single colony or ~30 population doublings. In normoxic incubators, however, cell proliferation diminished after ~3 such transfers. Optimal growth conditions in Medium $MH6^{+++}$ were identified through examination of approximately 60 variant growth regimens; parameters studied included plate-coating techniques (laminin, collagen, fibronectin) at different concentrations; media (DMEM hi/low glucose, NDiff RHB-A medium (Stem Cell Sciences), Knockout Medium (Gibco), Leibovitz's L-15 Medium, and DMEM-F12); CO_2 and O_2 concentrations; growth factor additions (bFGF, EGF, PDGF, IGF1, IGF2, VEGF); and frequency of medium-changing. For continuous growth of PrCa cells in Medium $MH6^{+++}$ the recombinant growth factors bFGF, EGF and bovine pituitary extract were essential.

Normal human prostate epithelial cells (NHuPrEC, lot 7F4228, 21 year old male) were purchased from Lonza (Walkersville Md) and from LifeLine Cell Technology (Frederick, MD) (lots #02321, 02383, 02490, from 20 yr, 25 yr, and 30 yr old males, respectively) and were grown in the same medium on laminin coated tissue culture wells.

For some orthotopic xenograft assays, cells were retrovirally infected to express Enhanced Green Fluorescent Protein (EGFP). Monolayer cultures were spinfected with viral supernatant and 10 μg/ml polybrene for 45 min at 900 RCF (Beckman TJ-6, TH-4 rotor) and incubated at 37°C for 20 min. Efficiency of infection was 30-50% by fluorescence microscopy.

Adherent-cell growth curves were determined by images taken at 0 h, 24 h, and 48 h after initial colony appearance and quantified by NIH ImageJ software [1]. Senescence-associated β-galactosidase was determined as described [2].

Generation of spheres in Matrigel cultures. Adherent PrCa cells were trypsinized to single cells, passed through a 40μm strainer and plated in 1 ml Matrigel cultures in medium $MH6^{+++}$ in 5 mg/ml (final) Matrigel. 10^4 cells in Matrigel were plated in 24-well cluster wells that were pre-coated with 0.5 ml 5mg/ml Matrigel in medium $MH6^{+++}$ to prevent cells from adhering to and growing on the well surface. Sphere cultures were replenished with 1 ml of $MH6^{+++}$ medium on top of the solidified Matrigel every 2 d. To harvest spheres grown in Matrigel suspension cultures, spheres and Matrigel were harvested on successive days of growth and treated for 60 min at 37°C with 1 mg/ml (final) Dispase I (Sigma). The spheres were carefully pelleted (1000xg, 12 min) , resuspended in PBS and carefully spun onto cleaned microscope slides in a

cytocentrifuge at 280 rpm for 12 min. In this way the spheres stay fully intact and the number of cells per sphere can be reliably counted and their growth kinetics followed. The growing spheres could be reliably shown to develop from single PrCa cells. The great majority (>90%) of adherent, early-passage single-cell PrCa cells give rise to spheres that grow continuously for 12-14 d, after which cells in the center of the spheres start to differentiate and proliferation of cells in PrCa-spheres decelerates. Dispase-harvested spheres were trypsinized, passed through 40 μm strainers, and replated in Matrigel culture up to three successive cycles. Figure 4 shows the growth of PrCa-derived spheres starting with single PrCa cells. With increasing transfers of the adherent PrCa cells, a smaller fraction of the adherent grew as spheres, and after having been transferred as adherent cells at a 1:3 split ratio 6-7 times, <0.1% of adherent PrCaCells generated spheres.

Flow Cytometry. Single or pooled colonies were sub-cultured in laminin-coated 12-well plates (Corning) in growth media until ~80% confluent. Samples were fixed in 2% paraformaldehyde for 20 min and in permeabilization buffer (eBiosciences) for high molecular weight cytokeratin CK5/14 (Dako, 34bE12) antibody staining, or stained live for CD133 (Miltenyi, AC133), integrin $\alpha 2\beta 1$ (Abcam, P1E6) or CD44 (Santa Cruz Biotech, IM7), and ALDH1 (Aldefluor, Stem Cell Technology). Antibody dilutions were used at the manufacturer's recommendations. Secondary antibodies used were 1:100 dilutions of anti-mouse-AlexaFluor488 (Invitrogen) and anti-rat-PE (Jackson ImmunoResearch Laboratories). Live cells were counterstained with propidium iodide.

Reverse Transcriptase Polymerase Chain Reaction (RT-PCR).

mRNA samples isolated by TRIZOL/Qiagen RNeasy columns were amplified by means of the Bioline (Taunton, MA) One-Step RT-PCR kits in a Thermocycler according to the manufacturer's directions and using the following temperature sequences. For amplifying TERT mRNA: 45°C (20 min), 95°C (1 min), 61°C (10 sec), 72°C (30 sec) 40 cycles, see also Supplementary Table S2. Amplification of TMPRSS2-ERG fusion mRNA: 45°C (20 min), 95°C (1 min), 71°C (10 sec, very stringent), 72°C (30 sec), 40 cycles. One-Step Amplification of the fusion mRNA was pursued by means of several different amplification oligo sets, as detailed in Supplementary Table S2. To amplify the TMPRSS2-ERG fusion mRNA, RNA extracted from the VCaP prostate cancer cell line was used as a fusion-mRNA positive control, using as little as 1 ng total RNA of the VCaP cell line. Amplified samples were run with 10μL of dye on 1.2% agarose gels in TAE buffer. Amplification products were purified, sequenced and compared to published human gene sequences to verify the identity of the products.

ALDEFLUOR Assay. Aldehyde dehydrogenase activity, a cytoplastic functional stem cell marker, was assayed by the fluorescence ALDEFLUOR assay kit (StemCell Technologies) following the manufacturer's protocol. In short, dissociated cells from PrCa cell cultures or from control cell lines were suspended in ALDEFLUOR assay buffer at 1.5 mM, which contains the ALDH1A1 substrate bodipy-aminoacetaldehyde (BAAA), and incubated for 60 min at 37°C. An inhibitor of ALDH, diethylaminobenzaldehyde (DEAB) used at a 12-fold molar excess served as a control for the specificity of ALDH enzyme detection. ALDEFLUOR-treated PrCa cells were run on BD FACSAria II Flow cytometer and bodipy fluorescence was analyzed by BD FACSDiva software V6.1.3 (BD Biosciences) and FlowJo software (TreeStar). To assay for the

expression of specific ALDH isotypes by PrCa cells, the cells were grown on laminin-coated coverslips, fixed (MeOH, -20°C, 10 min) and subjected to immunofluorescence with rabbit antisera specific for ALDH isotypes 3A1 and 7A3 (ABGENT, San Diego) and non-immune rabbit serum immunoglobulin as control. The expression by PrCa cells of specific ALDH isotypes among the 19 different known isotypes of ALDH was tested by immunocytochemistry, as detailed below.

Immunocytochemistry Cells grown on laminin-coated glass coverslips were fixed in cold MeOH for 10 min at -20°C, permeabilized in 0.5% TritonX-100, blocked in 5% donkey serum, and stained according to vendor's guidelines with antibodies described above to CK5/14, CD133, integrin $\alpha2\beta1$, and CD44, or additionally with antibodies to chromogranin A (Dako DAK-A3), p63 (Santa Cruz sc-8431, mAB 4A4), CK8/18 (Santa Cruz sc-6259, mAB DC-10), PSA (Genetex GTX28681, mAB ER-PR8), androgen receptor (Santa Cruz sc-7305, mAB 441), ERα (Abcam ab7820, mAB B401 (AER303)), ERβbeta (GeneTex GTX70174, mAB 14C8), c-kit (Santa Cruz sc-65276, mAB 57A5), Tra-1-81 (Cell Signaling 4745P, mAB Tra-1-81), SSEA4 (Cell Signaling 4755P, mAB MC813), E-Cadherin (Cell Signaling 3195P, mAB 24E10), Aldehyde dehydrogenase isotype 1A3 (ABGENT AP7847a) and ALDH isotype 7A1 (ABGENT AJ1002a) at 4°C overnight. Samples were stained with APC and FITC (BD Biosciences) or Alexa Fluor AF647 or AF568 (Invitrogen), or Jackson Immunoresearch 488-conjugated goat anti-rabbit IgG (H+L, #111-545-003), 488-conjugated Donkey anti-mouse IgG (H+L), or Jackson 488-conjugated donkey-anti-mouse IgM secondary antibodies, and counterstained with Pro Gold Antifade with DAPI (Invitrogen). Slides were imaged with a FluoView FV100 (Olympus) confocal microscope.

For immunohistochemistry of mouse sections, mice were sacrificed at 12-16 weeks post-implantation. Organs were fixed in 10% buffered neutral formaldehyde, blocks were produced and sectioned, and stained with hematoxylin-eosin. Sections were deparaffinized and stained with primary antibodies described above to CK5/14, CD133, CD44, and p63, or additionally with antibodies to Alpha-Methylacyl-CoA Racemase (Zeta Z2001,mAB 13H4), PSA (Dako IR514), ERα (Dako mAB 1D5), E-cadherin (Zymed 4A2C7), surface of intact human mitochondria (Millipore 113-1), and EGFP (Clontech #632375). Reaction products were developed by ABC system (VectorLabs). All mAB use was controlled with corresponding isotype control antibodies.

Orthotopic xenografting. Collagen grafts were prepared by resuspending the indicated number of cultured PrCa cells in neutralized rat tail collagen and incubating cells overnight in growth medium. SCID/Beige male mice (Charles River) or surgically *plus* chemically castrated SCID/Beige mice were anesthetized with ketamine/medetomidine according to approved protocols, and the urogenital organs were externalized. A single anterior prostate capsule was pierced with a 21-gauge syringe to release prostatic fluid, prior to implanting the collagen graft into the anterior prostate. In intact male mice a 25 mg dihydrotestosterone pellet was subcutaneously implanted adjacent to the incision site. Surgically castrated mice were used 2 weeks after surgical castration and were given 40 mg/kg bicalutamide (androgen antagonist) twice a week. *In vivo* imaging of EGFP fluorescence was carried out using the eXplore Optix system (GE). Control mice were transplanted with normal human prostate epithelial cells (NHPrEC) grown as described for PrCa cells and purchased from LONZA (lot 02321, 21-yr

donor) and LifeLine (lot 7F4228, 20-yr donor). NHPrEC from young donors were TERT-negative.

Tissue recombination and kidney capsule grafting. Female Harlan Sprague Dawley rats at 17.5 d gestation were sacrificed according to university-approved protocols and urogenital sinuses dissected from the embryos. Urogenital mesenchyme (UGM) was separated from urogenital epithelium after mild trypsinization at 4°C and microdissection. Single cells were obtained by treating UGM with collagenase at 37°C. To prepare tissue recombinants, $2.5x10^5$ UGM cells were combined with $1x10^5$ prostate epithelial cells in neutralized rat tail collagen. Collagen grafts were kept in growth media at 5% O_2 overnight. Grafts were implanted under the renal capsule of Severe Combined Immunodeficiency (SCID)/Beige male mice (Charles River). $2.5x10^5$ UGM cells in a collagen graft implanted as a negative control. Mice were supplemented with testosterone by subcutaneous insertion of a 25 mg dihydrotestosterone pellet and sacrificed at 12-16 weeks post-implantation. Negative control mice were observed and then processed 26 weeks after implantation.

Ethics Statement: All human samples were anonymously coded and obtained according to UCSD IRB#130397. Ethical use of immune deficient mice for the orthotopic transplantation of human prostate cancer cells has been authorized by the UCSD IACUC authorities by means of the animal protocol # S07410.
