# Supplementary material for: Early Human Prostate Adenocarcinomas Harbor Androgen-Independent Cancer Cells
Source: PLoS One. 2013 Sep 25;8(9):e74438. doi: 10.1371/journal.pone.0074438 (PMC3783414; doi:10.1371/journal.pone.0074438)
Supplement: Results S1 — (PDF) [file pone.0074438.s006.pdf]

SUPPLEMENTARY RESULTS S1:

UGM-Mediated Tissue Recombination. The biological activity of the PrCa cell cultures *in vivo* was assayed by tissue recombination with rat embryonic urogenital mesenchyme (UGM) and implantation under the kidney capsule of male SCID mice. Tissue recombination mimics the embryonic microenvironment in which the urogenital sinus develops into urinary and reproductive organs, and has been shown to enable human prostate cells to differentiate *in vivo* [3].

Tissue recombinants were prepared using $1x10^5$ prostate epithelial cells cultured as described above, combined with $2.5x10^5$ UGM cells. The resulting sub-renal cortex grafts were analyzed by standard histologic methods 12 weeks after grafting. Hematoxylin-eosin staining revealed the formation of glandular structures under recipient kidney capsules, usually composed of a single-layer of epithelium (Fig S4A). Expression of E-cadherin in subcapsular glands induced by PrCa cells confirmed an epithelial character of the cells (Fig S4B). A variable, usually high percentage of cells that lined the simple, cuboidal glands was positive for p63, a marker associated with the basal compartment and with putative prostate stem cells (Fig S4C) [4,5]. To confirm the human origin of the glandular structures, we showed that the glandular cells stained positive with a species-specific anti-human mitochondrial antibody (Fig S4D). The simple glands formed in this assay did not express Alpha-Methylacyl-CoA Racemase (data not shown), usually over-expressed by malignant human prostate tissue [6]. Unlike the expression profile *in vitro,* the observed glands were negative for CK5/14, CD44, and CD133 (data not shown). As controls for

the tissue recombination engraftment, when $2x10^5$ UGM cells were transplanted alone, no glands developed after 24 weeks.

In summary, when examined by tissue recombination engraftment with rat embryonic UGM and implantation under the kidney capsule of recipient mice, the cells cultured from human prostate adenocarcinoma samples did not appear to be tumorigenic during the observation period of 12 weeks. This suggests that the tissue recombination microenvironment imposed upon the human PrCa cells by the embryonic UGM cells led to apparently “normal” differentiation of the tumor-derived cells into structures resembling neonatal prostate, a finding described previously in another context [7,8].
