# Supplementary material for: Early Human Prostate Adenocarcinomas Harbor Androgen-Independent Cancer Cells
Source: PLoS One. 2013 Sep 25;8(9):e74438. doi: 10.1371/journal.pone.0074438 (PMC3783414; doi:10.1371/journal.pone.0074438)
Supplement: Table S1 — Culture of Representative Prostate Cancer Tissue Samples. (PDF) [file pone.0074438.s007.pdf]

Supplementary TABLE S1. Culture of Representative Prostate Cancer Tissue Samples.

Lab ID	Primary Gleason Grade	Secondary Gleason Grade	Gleason Score	Sample Histology	Number colonies from 50 mg dispersed tissue
3	3	4	7	Adenocarcinoma. High-grade PIN. Glandular hyperplasia.	4
4	4	3	7	Adenocarcinoma. High-grade PIN. Glandular atrophy.	68
6	3	4	7	Adenocarcinoma. BPH.	0
8	4	4	8	Adenocarcinoma. Extensive high-grade PIN.	0
9	4	5	9	Adenocarcinoma. High-grade PIN.	120
12	3	4	7	Adenocarcinoma. BPH. PIN. Stromal hyperplasia.	200
13	3	4	7	Adenocarcinoma. Extensive high-grade PIN.	260
14	3	4	7	Adenocarcinoma. High-grade PIN. Atrophy.	0
15	4	4	8	Adenocarcinoma. Basal cell hyperplasia.	0
22	3	3	6	Adenocarcinoma. Glandular and stromal hyperplasia.	0
27	3	3	6	Adenocarcinoma. Glandular hyperplasia. Stromal hyperplasia.	0
28	4	4	8	Adenocarcinoma. High-grade PIN.	0
31	3	3	6	Adenocarcinoma. Basal cell hyperplasia. Glandular and stromal hyperplasia.	0
48	3	4	7	Adenocarcinoma. High-grade PIN and BPH.	<5
52	4	3	7	Adenocarcinoma. Glandular hyperplasia and atrophy.	75
57	3	4	7	Adenocarcinoma. Focal glandular hyperplasia.	42
62	3	3	6	Adenocarcinoma. High-grade PIN. Stromal hyperplasia. BPH.	59
63	3	3	6	Adenocarcinoma. High-grade PIN. BPH.	48
66	4	4	8	Adenocarcinoma. High-grade PIN. Benign prostatic and stromal hyperplasia.	2
69	3	4	7	Adenocarcinoma. High-grade PIN. Glandular hyperplasia and atrophy.	250
73	4	5	9	Adenocarcinoma. High and low grade PIN. Glandular/stromal hyperplasia.	220
76	3	3	6	Adenocarcinoma. High and low grade PIN.	180
77	3	4	7	Adenocarcinoma	300
78	3	3	6	Adenocarcinoma	120
79	3	4	7	Adenocarcinoma	240
80	3	4	7	Adenocarcinoma	0
81	4	3	7	Adenocarcinoma. Small acinar type.	6
84	3	4	7	Adenocarcinoma	86
85	3	4	7	Adenocarcinoma	0
86	4	4	8	Adenocarcinoma	240
87	3	4	7	Adenocarcinoma	350
88	3	4	7	Adenocarcinoma	48
90	4	3	7	Adenocarcinoma	0
91	3	4	7	Adenocarcinoma	80
92	4	4	8	Adenocarcinoma. Conventional.	0
97	3	3	6	Adenocarcinoma	280
99	4	4	8	Adenocarcinoma	249
100	3	3	6	Adenocarcinoma	238
102	3	4	7	Adenocarcinoma	0
105	3	4	7	Adenocarcinoma	180
106	3	4	7	Adenocarcinoma	90
108	4	3	7	Adenocarcinoma	230
109	3	5	8	Adenocarcinoma	40
