# Supplementary material for: Early Human Prostate Adenocarcinomas Harbor Androgen-Independent Cancer Cells
Source: PLoS One. 2013 Sep 25;8(9):e74438. doi: 10.1371/journal.pone.0074438 (PMC3783414; doi:10.1371/journal.pone.0074438)
Supplement: Table S2 — Reverse Transcriptase PCR Condition. (PDF) [file pone.0074438.s008.pdf]

Supplementary TABLE S2. Reverse Transcriptase PCR Conditions.

Transcript/Gene	Primer Sequences	Ref Sequence	Product
Telomerase Reverse Transcriptase (TERT) [9]	Forward: 5'-CAGCTCCCATTTCATCAGCA-3' (nt 2921-2940)<br>Reverse: 5'-CGACATCCCTGCGTTCTTG-3' (nt 3034-3016)	TERT: NM_001193376.1	114 bp
GAPDH (control) [9]	Forward: 5'-CGACAGTCAGCCGCATCTT-3' (nt 118-136)<br>Reverse: 5'-TTCCCCATGGTGTCTGAGC -3' (nt 182-164)	GAPDH: NM_002046.4	65 bp
TMPRSS2-ERG Fusion [10-13]	Forward: 5'-TAGGCGCGAGCTAAGCAGGAG-3' (nt 4-24)<br>Reverse: 5'-GTAGGCACACTCAAACAACGACTGG-3' (nt 362-338)	TMPRSS2: NM_005656.3<br>ERG: NM_004449.4	126 nt
	Forward: 5'-CAGGAGGCGGAGGCGGA (nt 19-35)<br>Reverse: 5'-GGCGTTGTAGCTGGGGGTGAG-3' (nt 848-828)	TMPRSS2: NM_005656.3<br>ERG: NM_004449.4	597 nt
