# Supplementary material for: Early Human Prostate Adenocarcinomas Harbor Androgen-Independent Cancer Cells
Source: PLoS One. 2013 Sep 25;8(9):e74438. doi: 10.1371/journal.pone.0074438 (PMC3783414; doi:10.1371/journal.pone.0074438)
Supplement: References S1 — (PDF) [file pone.0074438.s009.pdf]

SUPPLEMENTARY REFERENCES S1:

1. Hueck IS, Haas M, Finones R, Frimodig J, Gough DA (2011) The potential of selectively cultured adult stem cells re-implanted in tissues. Stem Cell Engineering 1: 79-118.
2. Dimri GP, Lee X, Basile G, Acosta M, Scott G, et al. (1995) A biomarker that identifies senescent human cells in culture and in aging skin in vivo. Proc Natl Acad Sci U S A 92: 9363-9367.
3. Hayward SW, Haughney PC, Rosen MA, Greulich KM, Weier HU, et al. (1998) Interactions between adult human prostatic epithelium and rat urogenital sinus mesenchyme in a tissue recombination model. Differentiation 63: 131-140.
4. Senoo M, Pinto F, Crum CP, McKeon F (2007) p63 Is essential for the proliferative potential of stem cells in stratified epithelia. Cell 129: 523-536.
5. Signoretti S, Waltregny D, Dilks J, Isaac B, Lin D, et al. (2000) p63 is a prostate basal cell marker and is required for prostate development. Am J Pathol 157: 1769-1775.
6. Rubin MA, Zhou M, Dhanasekaran SM, Varambally S, Barrette TR, et al. (2002) alpha-Methylacyl coenzyme A racemase as a tissue biomarker for prostate cancer. Jama 287: 1662-1670.
7. Cunha GR, Hayashi N, Wong YC (1991) Regulation of differentiation and growth of normal adult and neoplastic epithelia by inductive mesenchyme. Cancer Surv 11: 73-90.
8. Wong YC, Cunha GR, Hayashi N (1992) Effects of mesenchyme of the embryonic urogenital sinus and neonatal seminal vesicle on the cytodifferentiation of the Dunning tumor: ultrastructural study. Acta Anat (Basel) 143: 139-150.

9. Beatty GL, Vonderheide RH (2008) Telomerase as a universal tumor antigen for cancer vaccines. Expert Rev Vaccines 7: 881-887.

10. Rajput AB, Miller MA, De Luca A, Boyd N, Leung S, et al. (2007) Frequency of the TMPRSS2:ERG gene fusion is increased in moderate to poorly differentiated prostate cancers. J Clin Pathol 60: 1238-1243.

11. Wang J, Cai Y, Ren C, Ittmann M (2006) Expression of variant TMPRSS2/ERG fusion messenger RNAs is associated with aggressive prostate cancer. Cancer Res 66: 8347-8351.

12. Mani RS, Tomlins SA, Callahan K, Ghosh A, Nyati MK, et al. (2009) Induced chromosomal proximity and gene fusions in prostate cancer. Science 326: 1230.

13. van den Hoogen C, van der Horst G, Cheung H, Buijs JT, Lippitt JM, et al. (2010) High aldehyde dehydrogenase activity identifies tumor-initiating and metastasis-initiating cells in human prostate cancer. Cancer Res 70: 5163-5173.
